# OR14-001 – Tocilizumab in autoinflammation and AA amyloidosis

**DOI:** 10.1186/1546-0096-11-S1-A268

**Published:** 2013-11-08

**Authors:** T Lane, JD Gillmore, AD Wechalekar, NM Stewart, JA Gilbertson, S Sachchithanantham, DM Rowczenio, S Banypersad, JH Pinney, S Mahmood, HJ Lachmann, PN Hawkins

**Affiliations:** 1 National Amyloidosis Centre, University College London, London, UK

## Introduction

The value of IL-6 blockade has been established in rheumatoid arthritis (RA) and systemic onset juvenile idiopathic arthritis (SOJIA), but its wider utility in patients with AA amyloidosis and autoinflammatory diseases has been little studied.

## Objectives

To assess clinical and serological responses to tocilizumab therapy in adult patients with AA amyloidosis and various autoinflammatory disorders.

## Methods

16 patients at the UK National Amyloidosis Centre with AA amyloidosis and various autoinflammatory disorders that were refractory to various treatments underwent therapeutic trials of tocilizumab. Disease activity and treatment response were monitored by symptoms, serial SAA and CRP measurements and a comprehensive range of standard blood and urine analyses. Amyloid load was evaluated and monitored by SAP scintigraphy.

## Results

13 (81%) patients had AA amyloidosis (7 RA, 3 JIA, 1 hyper IgD and periodic fever syndrome [HIDS], 1 presumed Castleman’s disease, and 1 unclassified autoinflammatory disorder), and 3 had severe, longstanding refractory autoinflammatory disorders (1 HIDS and 2 unclassified autoinflammatory disorders). 10 (63%) patients were male. Median age at presentation to our clinic was 48 years (inter-quartile range, IQR, 23-52). All patients had received at least one previous unsuccessful treatment with anti-cytokine or other disease modifying anti-rheumatic therapies prior to receiving tocilizumab.

Median SAA concentration prior to tocilizumab treatment, calculated as the median of all SAA measurements for each individual in the 12 months preceding initiation of therapy, ranged from 10 – 414mg/L; serum SAA concentration following introduction of tocilizumab was a median of 3mg/L for the entire cohort (IQR 3 – 6), i.e. indicating complete normalisation of SAA (Mann Whitney test p<0.0001). All patients reported improvement in symptoms. During median follow-up of 21 months (IQR 11–43), SAA values remained normal/near normal in 14 patients; median SAA concentration was 11mg/L and 18mg/L in the two remaining patients, representing substantial improvement from pre-treatment medians of 58mg/L and 198mg/L respectively. 8 patients, all of whom responded completely to tocilizumab, have had follow-up SAP scans, demonstrating regression of amyloid in 6 (75%) and stable deposits in 2 cases.

## Conclusion

Treatment with tocilizumab has been successful in producing sustained suppression of refractory inflammatory disease, both serologically and symptomatically, in 14 of 16 (88%) of patients treated under our care, with accompanying regression of amyloid deposits in 6 (75%) of these.

## Disclosure of interest

None declared

